# Study of Statin- and Loratadine-Induced Muscle Pain Mechanisms Using Human Skeletal Muscle Cells

**DOI:** 10.3390/pharmaceutics9040042

**Published:** 2017-10-10

**Authors:** Yat Hei Leung, Jacques Turgeon, Veronique Michaud

**Affiliations:** 1 Faculty of Pharmacy, Université de Montréal, Montreal, QC H2X 0A9, Canada; yat.hei.leung@umontreal.ca (Y.H.L.); turgeoja@gmail.com (J.T.); 2 Centre de Recherche du Centre Hospitalier de l’Université de Montréal (CRCHUM), Montreal, QC H2X 0A9, Canada

**Keywords:** statins, loratadine, drug-transporters, MCT, monocarboxylate transporters, lactic acid, skeletal muscle cell, drug-induced muscle disorders

## Abstract

Many drugs can cause unexpected muscle disorders, often necessitating the cessation of an effective medication. Inhibition of monocarboxylate transporters (MCTs) may potentially lead to perturbation of l-lactic acid homeostasis and muscular toxicity. Previous studies have shown that statins and loratadine have the potential to inhibit l-lactic acid efflux by MCTs (MCT1 and 4). The main objective of this study was to confirm the inhibitory potentials of atorvastatin, simvastatin (acid and lactone forms), rosuvastatin, and loratadine on l-lactic acid transport using primary human skeletal muscle cells (SkMC). Loratadine (IC_50_ 31 and 15 µM) and atorvastatin (IC_50_ ~130 and 210 µM) demonstrated the greatest potency for inhibition of l-lactic acid efflux at pH 7.0 and 7.4, respectively (~2.5-fold l-lactic acid intracellular accumulation). Simvastatin acid exhibited weak inhibitory potency on l-lactic acid efflux with an intracellular lactic acid increase of 25–35%. No l-lactic acid efflux inhibition was observed for simvastatin lactone or rosuvastatin. Pretreatment studies showed no change in inhibitory potential and did not affect lactic acid transport for all tested drugs. In conclusion, we have demonstrated that loratadine and atorvastatin can inhibit the efflux transport of l-lactic acid in SkMC. Inhibition of l-lactic acid efflux may cause an accumulation of intracellular l-lactic acid leading to the reported drug-induced myotoxicity.

## 1. Introduction

Adverse drug reactions (ADRs) are an important public health problem. Death caused by ADRs has increased over the years and, since 2011, has actually surpassed motor vehicle traffic-related injuries [1]. There are many factors that can contribute to this situation, such as polypharmacy in the aging population, drug-drug interactions, and interindividual genetic variability modulating the pharmacodynamics and pharmacokinetics of drugs inside the organism [2,3,4]. Many common medications can induce musculoskeletal disorders, while their incidence is still unclear due to the lack of clear definitions (e.g., under drug-drug interaction conditions). However, drug-related musculoskeletal disorders have been reported more frequently since the introduction into the market of widely prescribed lipid lowering drugs, such as fibrates and statins [5]. Drug-induced myopathies can range from mild myalgias to myopathies with weakness and severe life-threatening rhabdomyolysis. While mild myalgias are more or less tolerable, chronic myopathies can affect quality of life. Therefore, an early recognition of these ADRs is really important for the patients, since most of them are partially or completely reversible when the offending drug is substituted or the dose is adjusted [6,7].

Statins, 3-hydroxy-3-methylglutaryl-coenzyme A (HMG-CoA) reductase inhibitors, form the number one class of drugs prescribed in the United States for the prevention of cardiovascular disease [8]. However, muscle pain is a known side effect associated with statin treatment. Statin therapy is usually well tolerated, but muscle symptoms can limit treatment adhesion or even lead to its discontinuation [9]. The definitive mechanism of statin-induced muscle disorders is still not known, although different hypotheses have been proposed, including alteration in cellular membrane cholesterol, alterations in protein synthesis and degradation, cell apoptosis, immune reactions, increased lysosomal activity, injuries to electrolytes homeostasis, inhibition of myogenesis, mitochondrial impairments, and oxidative stress [10,11,12].

Muscle is one of the largest human organs, and it is well-perfused, which means that it is also highly exposed to circulating drugs, making it quite susceptible to ADRs [13]. Since skeletal muscle is the major producer of l-lactic acid, the transport of l-lactic acid is critical for the maintenance of intracellular pH and homeostasis. We have previously hypothesized that drug-induced myotoxicities can be caused by an excess intracellular level of l-lactic acid. Indeed, we demonstrated that some statins are able to inhibit the efflux of l-lactic acid *via* MCT1 and MCT4 in breast cancer cell lines (Hs578T selectively expressing MCT1 and MDA-MB-231 selectively expressing MCT4). In those studies, atorvastatin and loratadine were associated with the greatest inhibitory potential on the efflux of l-lactic acid, leading to intracellular accumulation of lactic acid using cancer cells [14].

The main objective of our study was to corroborate our previous findings in physiologically relevant settings. Therefore, we proposed to characterize the effects of atorvastatin, loratadine, simvastatin lactone, simvastatin hydroxy acid, and rosuvastatin, on the transport of l-lactic acid using human skeletal muscle cells (SkMCs) at resting pH of 7.4 and at pH 7.0. A more acidic pH value was tested, since evidence suggests that drug-related muscle disorders can be exacerbated by exercise.

## 2. Materials and Methods

### 2.1. Materials

[^14^C] l-lactic acid sodium salt was purchased from PerkinElmer (Walthman, MA, USA). l-lactic acid sodium salt was obtained from Sigma-Aldrich (St. Louis, MO, USA). Atorvastatin, loratadine, phloretin, rosuvastatin, simvastatin, and simvastatin hydroxyl acid ammonium salt were purchased from Toronto Research Center (Toronto, ON, Canada). Cryopreserved human primary skeletal muscle cells (from adult), Human Skeletal Muscle Cells Growth medium, Human Skeletal Muscle Cells Differentiation medium, and Subculture Reagent Kit were purchased from Cell Applications Inc. (San Diego, CA, USA).

### 2.2. Cell Culture

**Figure 1:**
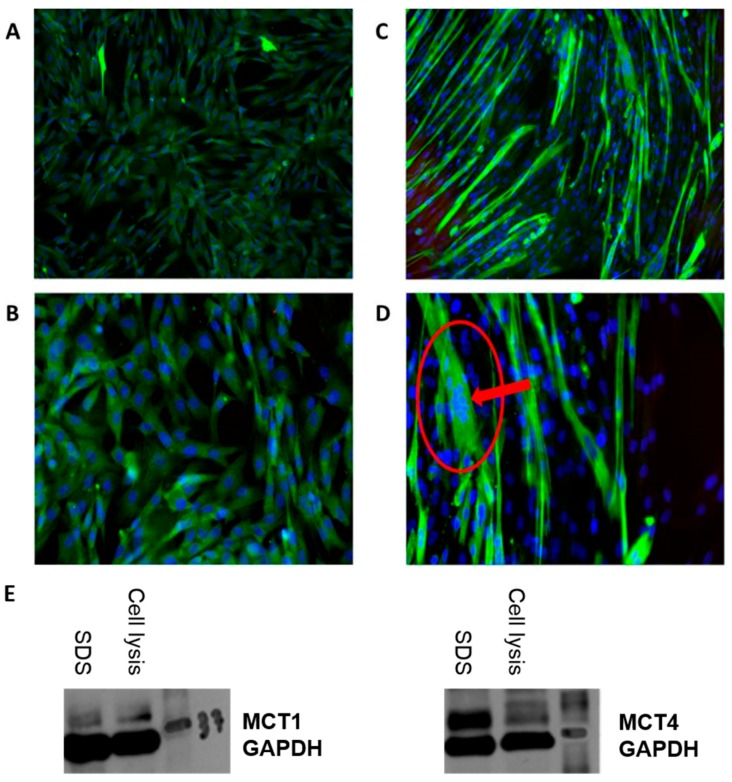
*SkMC*. Primary human myoblasts from Cell Applications Inc. were proliferated in 35 × 10 mm culture plates with SkMC growth medium for 25–35 days until 80–90% confluency and photographed at (**A**) 10× and (**B**) 20×. *SkMC in differentiation*. Differentiated SkMCs with multinucleated syncytia after exposition for 6 days with the SkMC differentiation medium are photographed at (**C**) 10× and (**D**) 20×. (**E**) Expression of MCT1 (**left**) and MCT4 (**right**) proteins in SkMCs was revealed by Western blotting using antibodies against MCT1, MCT4 and GAPDH (the 2 wells illustrated—SDS vs. cell lysis—represent 2 different methods tested for protein extraction).

SkMCs were grown in all-in-one-ready-to-use Human Skeletal Muscle Cells Growth medium and were used within 5 passages or 15 population doublings after thawing upon arrival or from storage in liquid nitrogen. Cells were first cultured in plastic culture flasks (Sarstedt, Newton, NC, USA) at 37 °C with 5% CO_2_. When they reached 60–80% confluence, they were harvested with Subculture Reagent Kit which includes HBSS, Trypsin/EDTA and Trypsin Neutralizing Solution, resuspended and seeded into new flasks. When the adequate amount of cells for the experiments was attained, they were again harvested, seeded on 35 × 10 mm tissue culture plates, and grown to reach 80–90% confluence before differentiation. After that, differentiation was initiated by changing the media from the Human Skeletal Muscle Cells Growth medium into the Human Skeletal Muscle Cells Differentiation medium for 6 days until the cells formed multinucleated syncytia, as seen in Figure 1A–D. The differentiation was confirmed by immunomicroscopy for expression of myosin (Skeletal, Slow) described in the next section.

### 2.3. Immunomicroscopy

The immunomiscroscopy images were obtained by having the SkMCs grown and differentiated on a glass slide cover. After removing the differentiation medium (or growth medim, if observations were made at an earlier stage), SkMCs were fixed with 3.7% formaldehyde for 15 min at room temperature and were washed twice with PBS 1× between every subsequent step. Samples were then quenched with glycine for 5 min, permeabilized with 0.2% Triton X-100 for 20 min and incubated for 3 h with BSA 3%. Cells were then incubated overnight at 4 °C with Monoclonal Anti-Myosin (Skeletal, Slow) antibody produced in mouse (Sigma-Aldrich, St. Louis, MO, USA). Cells were incubated with Alexa Fluor 488 (Invitrogen, Carlsbad, CA, USA) in BSA 3% for an hour at room temperature, followed by incubation with Hoechst for 15 min. After a final wash, images were acquired using an EE (×10 and ×20) and Zen Imaging software. The expression of MCT1 and MCT4 proteins has been assessed by Western blot during the cell culture optimization step (Figure 1E) (details of Western blotting are described in Supplementary Materials).

### 2.4. Transport Studies

After differentiation, and before the beginning of the transport experiments, cells were washed, and the medium replaced with HEPES (pH 7.4; 1 mL) buffer. Transport assays, including the pre-incubation period in HEPES, were performed at 37 °C. Assay conditions were previously optimized by standard incubations with l-lactic acid (e.g., incubation times). For the time-course experiments, different time points ranging from 5 s to 30 min were tested using one concentration (6 mM) of l-lactic acid.

Assessment of influx transport. At the beginning of the experiment (*t* = 0), HEPES medium was replaced by MES or HEPES (pH 7.0 or 7.4; 1 mL) buffer containing 0.03 to 30 mM [^14^C] l-lactic acid (0.2 µCi/mL). After incubation for 2.5 min, the radioactive media was removed from the milieu. Transport assays were stopped by placing culture plates on ice, rapidly aspirating the media and cells were washed 3-times with ice-cold HEPES buffer. Cells were then solubilized using a solution of 0.2 N NaOH and 1% SDS (500 µL). The suspension was passed through 27½ G needle 3-times. Aliquot of the cell lysate (400 µL) was transferred in a scintillation tube containing 5 mL of biodegradable scintillation counting cocktail buffer (Bio-Safe II, Research Products International Corp., Mt. Prospect, IL, USA). Radioactivity levels were quantified with a Tri-Carb liquid scintillation counter (LSC 1600TR, Packard Instrument Co., Meriden, CT, USA) to determine the intracellular [^14^C] l-lactic acid concentrations. Protein concentrations were measured using Pierce BCA protein assay kit (Thermo Fisher Scientific, Walthman, MA, USA).

Assessment of efflux transport. A similar approach was used to measure efflux transport of l-lactic acid. For these experiments, distribution equilibrium of l-lactic acid was reached by adding MES or HEPES (pH 7.0 or 7.4; 1 mL) containing 6 mM [^14^C] l-lactic acid (0.2 µCi/mL). After 10 min (to allow l-lactic acid to reach equilibrium), MES or HEPES buffer containing [^14^C] l-lactic acid was replaced by l-lactic acid-free buffer for 2.5 min. Transport was stopped by placing culture plates on ice, rapidly aspirating the media and cells were washed 3-times with ice-cold HEPES buffer. Cellular radioactivity levels were determined as previously described in the Method section (Assessment of influx transport).

### 2.5. Inhibition Studies

In the inhibition experiments, efflux of l-lactic acid was assessed in the presence and absence of increasing concentrations of statins and loratadine. In total, 5 compounds were investigated: atorvastatin, loratadine, rosuvastatin, simvastatin hydroxy acid, and simvastatin lactone. Effects of acidic drugs on l-lactic acid transport were tested at concentrations varying from 0.25 to 300 μM. Phloretin (1 to 300 µM) was also evaluated as a known potent MCT inhibitor.

At the beginning of the experiment (*t* = 0), medium was replaced by HEPES (pH 7.4; 1 mL) buffer to wash cells. After removing the wash buffer, cells were loaded with [^14^C] l-lactic acid (6 mM, 0.2 µCi/mL) in MES or HEPES buffer (pH 7.0 or pH 7.4). Briefly, following pre-incubation of cells in buffer containing [^14^C] l-lactic acid for 10 min (to allow l-lactic acid to reach equilibrium), the buffer was replaced by a buffer containing [^14^C] l-lactic acid and the potential inhibitor. This mixture was incubated for an additional 3 min in order to allow diffusion of the inhibitor in the cells without perturbing l-lactic acid equilibrium. Then, cells were washed once rapidly with buffer containing the tested inhibitors or vehicle, but without l-lactic acid. In the final step, the tested inhibitors or vehicle were added to the l-lactic acid-free buffer and incubated for 2.5 min as described previously for the assessment of the efflux transport. Culture plates were placed on ice to stop the reaction. Inhibition of l-lactic acid efflux was determined by measuring intracellular [^14^C] l-lactic acid concentration.

### 2.6. Effect of Pretreatment on Lactic Acid Transport

After differentiation, the cells were put in growth media for 24 h. After stabilization, cells were exposed to atorvastatin, simvastatin acid or loratadine (added to the media in DMSO) at clinically relevant concentrations (0.033 and 0.1 µM for atorvastatin, 0.033 and 0.1 µM for simvastatin acid, and 0.023 and 0.07 µM for loratadine) for six days before conducting transport experiments. Separate experiments were thereafter carried out as described previously in the *Transport Studies* and *Inhibition Studies* sections.

### 2.7. Quantification of Intracellular Concentrations of Statins and Loratadine

HPLC-UV methods were used to quantify atorvastatin, loratadine, rosuvastatin, simvastatin hydroxyl acid, and simvastatin lactone in the intracellular compartment of the cells. Instruments used consisted of a SpectraSystem P4000 pump, a SpectraSystem AS3000 autosampler, a Finnigan SpectraSystem UV6000 ultraviolet detector and a SpectraSystem SN4000 System Controller from Thermo Electron Corporation (San Jose, CA, USA). An Agilent Zorbax Column, Eclipse XDB-C8, 4.6 mm × 150 mm (Agilent, Santa Clara, CA, USA) was used at a temperature of 40 °C. An isocratic mobile phase contained 10 mM ammonium formate pH 3 and acetonitrile with varying proportions, at a flow rate of 1.0 mL/min. Details for mobile phase proportions, internal standards and monitored UV wavelengths are listed in Supplementary Table S1.

The same protocol as described in the *Inhibition Studies* section was used to measure intracellular concentrations of statins and loratadine at the end of the experiments, but without radioactive product (cold l-lactic acid). After the final incubation, cells were washed twice with PBS 10% methanol and once with PBS alone. The cells were lysed with methanol containing appropriate internal standards for the compounds of interest, then transferred and centrifuged for 10 min at room temperature. The supernatant was transferred to a culture borosilicate glass tube, evaporated and reconstituted in 100 µL of 10 mM ammonium formate pH 3 and acetonitrile (50:50 *v*/*v*). A volume of 20 µL per sample was injected. The ChromQuest Version 4.2.34 software was used for data acquisition.

### 2.8. Data Analysis

For kinetic studies, the K_m_ (Michaelis-Menten constant) and V_max_ (maximum uptake rate) of l-lactic acid transport were estimated by non-linear least-squares regression analysis program, GraphPad Prism 5.01 (GraphPad Software Inc., La Jolla, CA, USA) using the following equation:

*v* = V_max_·[S]/(K_m_ + [S]) (1)

where *v* and [S] are uptake rate of l-lactic acid at 2.5 min and concentration of l-lactic acid, respectively. CL_int_ and IC_50_ were also estimated using the GraphPad program (Version 5.01). For the IC_50_ determination, the intracellular level of l-lactic acid measured at the end of the 10-min pre-incubation period (when equilibrium was reached, and before adding the inhibitor) was considered as the reference of the maximal intracellular concentration attained beforehand to inhibit the efflux transport.

## 3. Results

### 3.1. Kinetic Parameters of l-Lactic acid Transport in SkMC

**Figure 2:**
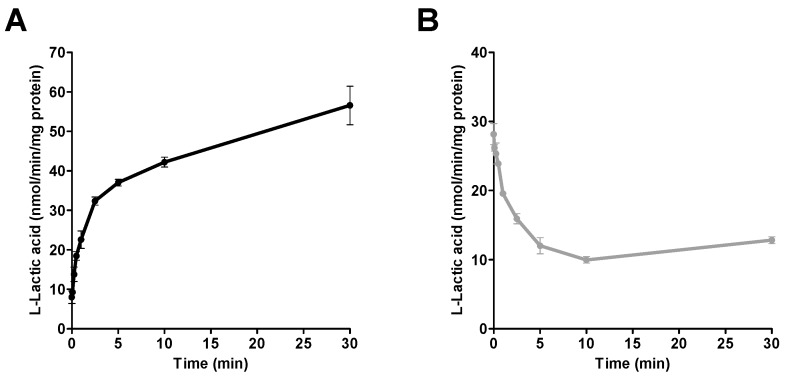
Intracellular concentrations of [^14^C] l-lactic acid over time: (**A**) the uptake of l-lactic acid (6 mM), and (**B**) the efflux of l-lactic acid in SKMC at an extracellular pH of 7.4. Each point represents the mean ± S.D. of experiments performed in triplicate.

The time-course for the uptake and efflux of l-lactic acid (6 mM) into SkMC was determined (Figure 2). The uptake and efflux of l-lactic acid were linear for the first minute while displaying a plateau thereafter. Those processes were rapid and most of the transport was completed within five minutes.

**Figure 3:**
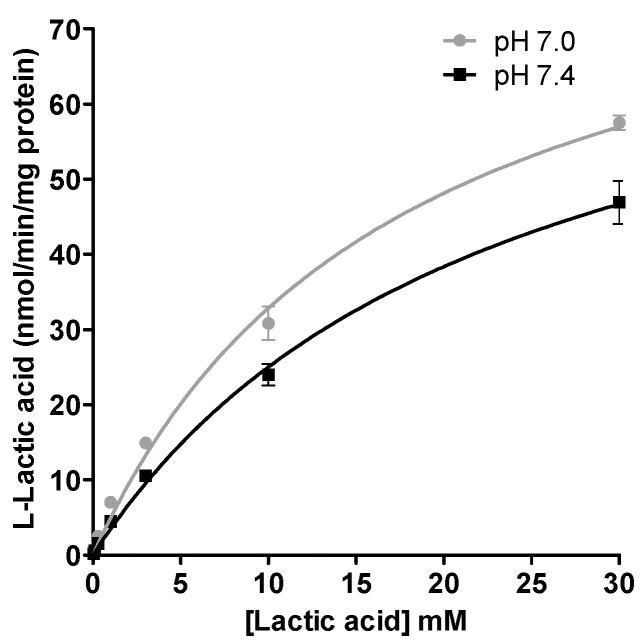
Kinetic parameters of l-lactic acid (0.03 to 30 mM) in SKMC determined at pH 7.0 and pH 7.4. Each point represents the mean ± S.D. of experiments performed in triplicate.

The kinetic parameters of the l-lactic acid influx transport in human skeletal muscle cells are illustrated in the Figure 3. The estimated CL_int_ value for the transport of l-lactic acid in SkMCs was higher at pH 7.0 than at pH 7.4; CL_int_ values were 5.2 µL/min/mg protein (V_max_ 90 nmol/min/mg protein; K_m_ 17 mM) vs. 3.6 µL/min/mg protein (V_max_ 82 nmol/min/mg protein; K_m_ 23 mM), respectively (Figure 3). The intrinsic clearance could be determined only for the influx transport.

### 3.2. l-Lactic Acid Efflux Inhibition by Different Drugs

**Figure 4:**
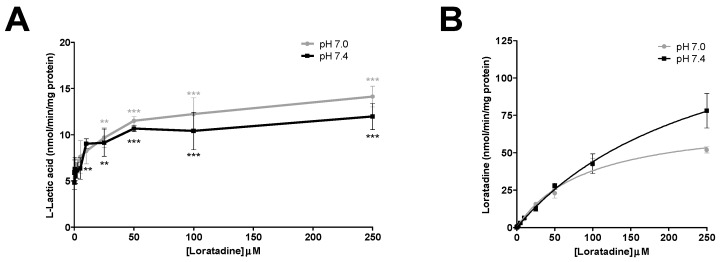
Drug inhibition studies with loratadine in SkMC. (**A**) Inhibitory effects of loratadine on l-lactic acid (6 mM) efflux in SkMC. The residual intracellular [^14^C] l-lactic acid was measured after 2.5 min of efflux at pH 7.0 and pH 7.4. (**B**) Intracellular concentrations of loratadine at the end of inhibition assays in SkMC at pH 7.0 and pH 7.4. Each point represents the mean ± S.D. of experiments performed in triplicate (* *p* < 0.05, ** *p* < 0.01 and *** *p* < 0.001).

**Figure 5:**
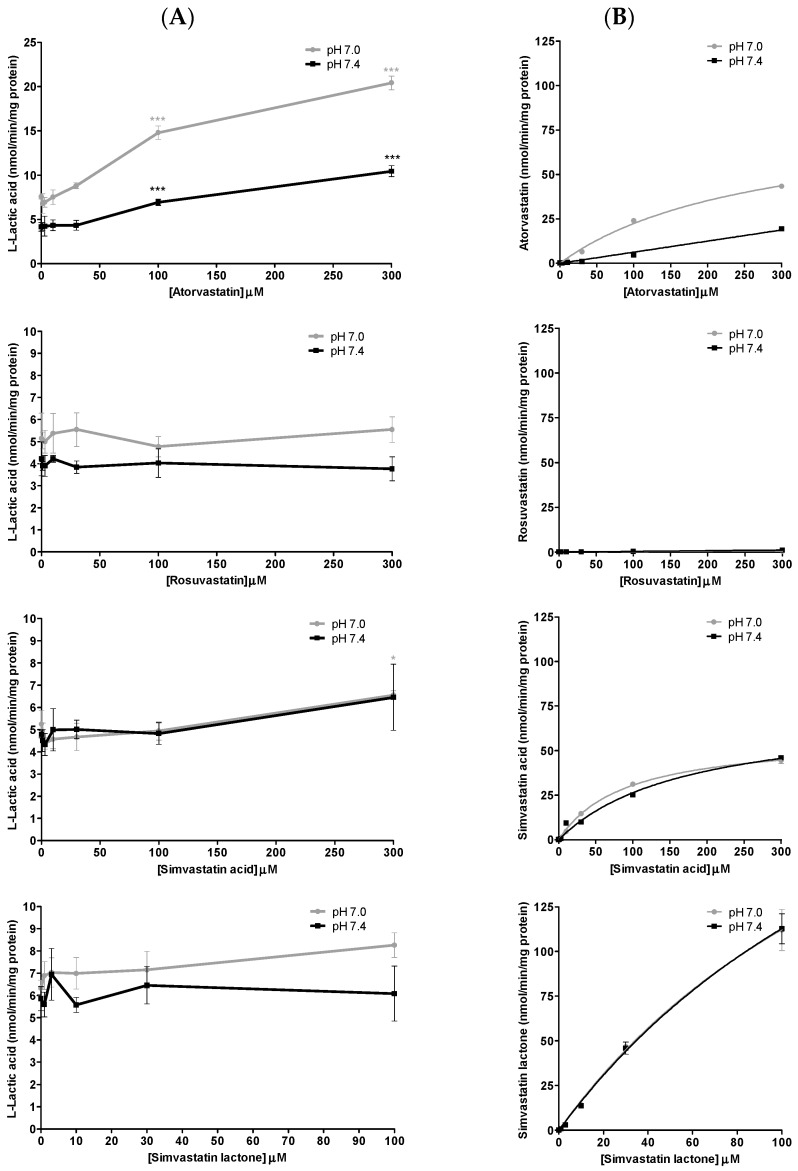
Drug inhibition studies with statins in SkMC. (**A**) Inhibitory effects of different statins (atorvastatin, rosuvastatin, simvastatin hydroxy acid and simvastatin lactone) on l-lactic acid (6 mM) efflux in SkMC. The residual intracellular [^14^C] l-lactic acid was measured after 2.5 min of efflux at pH 7.0 and pH 7.4. (**B**) Intracellular concentrations of statins (atorvastatin, rosuvastatin, simvastatin hydroxy acid and simvastatin lactone) at the end of inhibition assays in SkMC at pH 7.0 and pH 7.4. Each point represents the mean ± S.D. of experiments performed in triplicate (* *p* < 0.05, ** *p* < 0.01 and *** *p* < 0.001).

**Figure 6:**
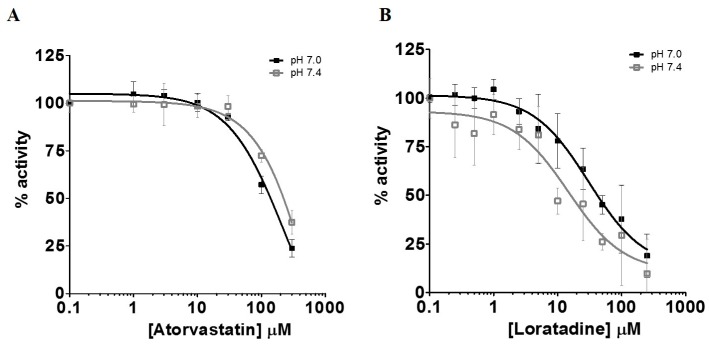
Inhibition of MCT-mediated efflux transport of l-lactic acid measured by the intracellular accumulation of l-lactic acid in the presence of atorvastatin (**A**) or loratadine (**B**). IC_50_ were determined at pH 7.0 and 7.4. The percentage of remaining activity was derived by substracting the maximal level of l-lactic acid after equilibrium to the residual intracellular l-lactic acid concentrations at the end of the experiment.

The inhibition of l-lactic acid efflux by statins and loratadine was tested at pH 7.0 and pH 7.4. In addition to pH 7.4, a more acidic pH value was assessed in order to determine whether an intense physical effort resulting in a lowered pH may modulate the inhibitory potential of the different compounds compared to a physiological pH. Figure 4A and Figure 5A present the intracellular accumulation of l-lactic acid in presence of increasing concentrations of potential inhibitors, i.e., loratadine and statins, respectively. Among the drugs tested, loratadine and atorvastatin had the highest inhibitory potential on the efflux of l-lactic acid. The intracellular l-lactic acid increased 2.5-fold in the presence of 250 µM loratadine at pH 7.4 compared to the control (Figure 4A). Similarly, at the highest tested concentration of 300 µM of atorvastatin, intracellular l-lactic acid was increased by 2.5-fold (Figure 5A). For simvastatin acid (300 µM), the maximal increase of intracellular l-lactic acid was only 35%. No significant inhibitory effect on the efflux transport of l-lactic acid was observed with simvastatin lactone and rosuvastatin (Figure 5A). The IC_50_ values were estimated for the most potent inhibitors of l-lactic acid efflux observed in our study (i.e., atorvastain and loratadine) (Figure 6A,B). Our results showed that, at pH 7.4, loratadine was a more potent MCT inhibitor than atorvastatin on the l-lactic acid efllux, with IC_50_ values of 15 µM and 210 µM, respectively).

The pH value had an effect on the basal activity of lactic acid transport. The accumulation of l-lactic acid in the SkMC was higher at pH 7.0 compared to pH 7.4. However, a similar magnitude of inhibition with statin on l-lactic acid transport was observed at pH 7.0 and pH 7.4. Our results showed a 2.7-fold increase in the intracellular concentration of l-lactic acid by atorvastatin 300 µM at pH 7.0 (vs. 2.5-fold at pH 7.4). Similar observations were made with loratadine, which caused similar efflux transport inhibitions of l-lactic acid at pH 7.0 vs. 7.4 (l-lactic acid intracellular concentrations increased by 2.3- vs. 2.5-fold, respectively). Again, under these conditions, simvastatin, lactone and rosuvastatin had no significant inhibitory effect on the transport of l-lactic acid.

### 3.3. Uptake of Different Drugs during Lactic Acid Efflux Inhibition

Figure 4B and Figure 5B illustrate the intracellular concentrations of the tested potential inhibitors, namely, loratadine and statins, respectively. Our results showed a higher accumulation of atorvastatin in SkMC at pH 7.0 compared to pH 7.4. Overall, pH values did not affect the intracellular penetration of simvastatin and loratadine (except at supratherapeutic concentration). Furthermore, rosuvastatin did not have a significant uptake in SkMC.

### 3.4. Validation of l-Lactic Acid Efflux Inhibition Using a Known Potent MCT Inhibitor

**Figure 7:**
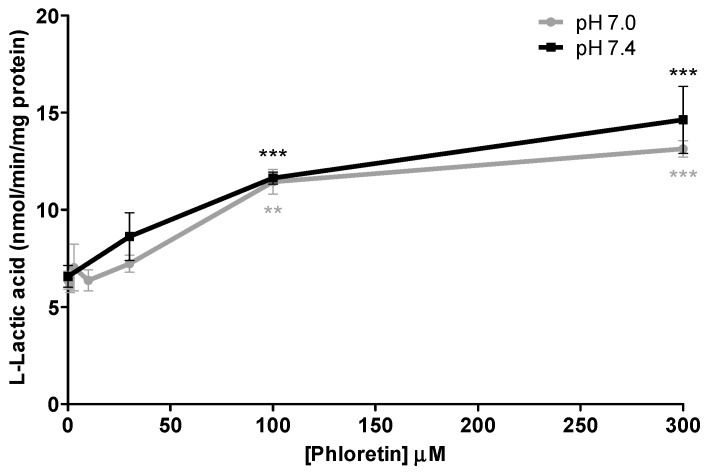
Inhibitory effects of phloretin, a known MCT inhibitor, on l-lactic acid efflux in SkMC. The intracellular [^14^C] l-lactic acid was measured after 2.5 min of efflux at pH 7.0 and pH 7.4 (** *p* < 0.01 and *** *p* < 0.001).

In order to compare the relative potency of the inhibition on l-lactic acid efflux via MCTs obtained with loratadine and statins, inhibition assays were also conducted with phloretin, a potent known MCT inhibitor. As shown in Figure 7, phloretin produced a maximal intracellular l-lactic acid augmentation of 2.1- and 2.2-fold at pH 7.0 and 7.4, respectively, which was similar to the observed inhibition with loratadine or atorvastatin. These results also indicated that the extent of inhibition of phloretin on MCTs was not affected by the pH tested.

### 3.5. Study of Pretreatment with Potential Inhibitors on l-Lactic Acid Transport in SkMCs

The three drugs with the highest potential inhibition (i.e., loratadine, atorvastatin and simvastatin hydroxy acid, based on prior data) of l-lactic acid efflux were selected for this study. Pretreatments with 0.033 µM and 0.1 µM of atorvastatin, 0.033 µM and 0.1 µM simvastatin acid, and 0.023 µM and 0.07 µM of loratadine were done to assess the transport capacity of l-lactic acid in SkMCs; these concentrations were selected based on clinically relevant concentrations, the highest tested concentrations were based on the maximal plasma concentrations for each substrate. Table 1 and Table 2 present the effects of various pretreatments with loratadine, atorvastatin or simvastatin hydroxy acid on the basal l-lactic acid transport, both influx and efflux transport were evaluated. Our first observation was that the basal influx activity of l-lactic acid transporters did not change following a pretreatment with either of these drugs (CL_int_ at pH 7.0 vs. 7.4) as seen in Table 1. Our second observation was that pretreatment has no significant effect on the inhibition by statins and loratadine on l-lactic acid efflux transport, as shown in Table 2.

## 4. Discussion

Our previous studies demonstrated, using cell lines expressing selectively high levels of MCT1 (Hs578T) or MCT4 (MDA-MB-231), that certain acidic drugs inhibit the efflux of l-lactic acid via monocarboxylate transporters [14]. These breast cancer cell line models are great tools for rapidly screening drugs that can potentially cause an intracellular accumulation of l-lactic acid and lead to the observed muscular symptoms. However, the use of these cell lines has some limitations. First, they have a higher MCT expression due to their higher need of energy and metabolism to support their great capacity to proliferate. Second, they are not the most physiologically representative type of cells for studying muscles. To corroborate our previous findings in a more physiologically representative model, we proposed the use of primary SkMCs to confirm the effects of statins and loratadine on l-lactic acid transport.

Skeletal muscles are the major producers of l-lactic acid in the body. Therefore, it is essential that l-lactic acid transporters maintain pH homeostasis, especially during physical effort, where more l-lactic acid is formed. It was reported that physically active patients were more susceptible to experiencing drug-induced muscle disorders [15,16,17,18]. The reason and mechanisms underlying this association are not well known; although it has been postulated that coenzyme Q10 deficiency due to statin administration could lead to impaired mitochondrial energy metabolism in muscle cells, the results are still controversial [15,16]. Other hypotheses indicate that the ubiquitin proteasome pathway (UPP), involved in cell degradation and repair, or sarcoplasmic reticulum calcium cycling could be altered by statin therapy [16]. However, another proposed mechanism for drug-induced myopathies involves l-lactic acid transport. Our hypothesis and results are also supported by previous observations indicating that statins could inhibit l-lactic acid transport, causing its intracellular accumulation [19]. It could also be speculated that this effect is mediated by co-transport of statins and l-lactic acid by MCTs, leading to competitive inhibition of the transporters.

Primary SkMCs were used in our experiments as an in vitro model of the actual muscle in order to study drug-induced myopathies. Among the statins tested, only atorvastatin (IC_50_ of 130–210 µM) and simvastatin hydroxy acid (35% increases in lactic acid intracellular levels at 300 µM) were found to be significant l-lactic acid efflux inhibitors. It is important to note that the inhibitory potency of l-lactic acid transport observed for atorvastatin was similar to that of the well characterized MCT inhibitor, phloretin.

As indicated previously, our results corroborate our previous findings, as well as other studies by Kobayashi et al., which showed that some statins—mainly the lipophilic ones, such as atorvastatin and simvastatin acid—can inhibit l-lactic acid transport via MCT4 [14,19]. In their model, they reported greater inhibitory potential for l-lactic acid uptake than the one we observed for its efflux. The differences between the results can be explained by the fact that we measured efflux inhibition, whereas they measured uptake inhibition. Furthermore, they used a much lower l-lactic acid concentration (3.3 µM) than the one used for our experiments (6 mM), as well as different cell models [19,20,21,22].

Our results are also in agreement with clinical data (Primo study), in which patients experienced muscular discomfort at a higher rate for statins with greater lipophilicity, such as atorvastatin (14.9%) and simvastatin (18.9%) [23]. Clinically, the higher frequency observed with simvastatin could be due to a greater propensity for drug-drug interactions, since simvastatin has a very low oral bioavailability (<5%) and greater potential for important increases in its exposure [24,25].

Loratadine, an H1 histamine antagonist, has also been reported to cause muscle pain. It is therefore possible that this antihistaminic may cause muscular toxicity through similar mechanisms as statins. Loratadine was determined to be the most potent l-lactic acid efflux inhibitor in this study, with an average IC_50_ of 15 µM at pH 7.4. Since this drug can be obtained without prescription, it is more difficult to estimate the true frequency of loratadine-induced ADR. In the literature, it was reported that loratadine was associated with an increased risk of myopathy in some drug combinations. However, data suggests that these drug interactions do not involve inhibition of its metabolism [26]. It is hypothesized that the interaction might occur at the muscular cellular level. It has been reported that the combination of loratadine and simvastatin is associated with an increased risk for myopathy (RR = 1.69) [27].

We also investigated the effect of pH on the inhibitory potential of some drugs on l-lactic acid transporters. A pH 7.4 milieu was used as the physiological pH level and pH 7.0 was selected as a representative post-exercise physiological condition, since it is known that patients who are more physically active are generally more susceptible to these drug-induced muscular disorders. Overall, we could not demonstrate an increased potency in the blocking of l-lactic acid efflux with a more acidic pH. It could be suggested that a decrease in pH should favor intracellular accumulation of statins or loratadine due to their biophysical properties and passive diffusion. Indeed, we observed an increase in the intracellular concentrations of atorvastatin under a more acidic pH. A trend was observed between the IC_50_ values of loratadine and atorvastatin and their respective intracellular concentrations. A lower IC_50_ was estimated for loratadine at pH 7.4, where its concentrations tended to be higher. For atorvastatin, the higher intracellular concentrations were observed at pH 7.0 and associated with lower IC_50_ for the efflux of l-lactic acid.

The use of primary human skeletal muscle cells also has some limitations. First, SkMCs take an extended period of time to grow and to produce the number of cells needed for the experiment. Second, it takes about one month for each batch of cells to reach maturity, which can impose an inter-batch variability. Moreover, the proportion of differentiated cells may vary for different batches, which could lead to differences in observed basal lactic acid transport activity.

In order to study the effects of statins or loratadine on skeletal muscle during a prolonged period of time, we pretreated the SkMCs with clinical concentrations of atorvastatin, simvastatin and loratadine for 6 days. The results showed that pretreatment with these drugs did not affect l-lactic acid transport activity. Pretreatment with these drugs did not affect their inhibitory potential either. Pre-exposure periods beyond 6 days were not recommended, because of the limited amount of time for which the cells could be kept in culture after differentiation. Additionally, we performed pretreatment assays at higher concentrations to assess the effect of short-term statin treatment on mRNA transporter expression in SkMCs (Supplementary Figure S1). No difference was observed in MCT1 and MCT4 expression between the control and the pretreated batches, which could explain the absence of change in l-lactic acid transport activity levels following pre-exposition to the drug.

In conclusion, we have developed a cell model that can be used to screen for different drugs that may contribute to drug-induced myopathy by inhibiting l-lactic acid efflux. Our experiments determined the inhibitory potential of different statins and loratadine on the transport of l-lactic acid by MCTs in human skeletal cells. Our results demonstrated that loratadine and atorvastatin blocked l-lactic acid efflux transport to a significant extent, and that the magnitude of this effect was not affected by pH variation during physical activity. However, there was a higher basal accumulation of l-lactic acid at pH 7.0 vs pH 7.4. Further studies are required to relate intracellular accumulation of l-lactic acid in skeletal muscle cells and the clinical observation of drug-induced muscle pain.

## Figures and Tables

**Table 1:** Kinetic parameters of l-lactic acid influx following a six-day pretreatment with atorvastatin, simvastatin hydroxy acid and loratadine in SkMC at pH 7.0 and pH 7.4.

Compound	Concentration	CL_int_ (nL/min/mg Protein)	
		pH 7.0	pH 7.4
Atorvastatin	Control	5.2	3.6
	0.033 µM	4.1	3.7
	0.1 µM	4.4	2.7
Simvastatin hydroxy acid	Control	4.5	2.3
	0.033 µM	3.6	2.5
	0.1 µM	2.4	1.5
Loratadine	Control	4.5	2.3
	0.023 µM	3.4	2.4
	0.07 µM	5.3	2.4

**Table 2:** Intracellular l-lactic acid increase (%) during l-lactic acid efflux inhibition studies following a six-day pretreatment with atorvastatin, simvastatin hydroxy acid and loratadine in SkMC at pH 7.0 and pH 7.4.

Compound ^1^	Concentrations Used for the Pretreatment	Intracellular l-Lactic Acid Increase (%)	
		pH 7.0	pH 7.4
Atorvastatin	Control	324	201
	0.033 µM	178 *	306 *
	0.1 µM	258	248
Simvastatin hydroxy acid	Control	91	65
	0.033 µM	103	51
	0.1 µM	31	35
Loratadine	Control	260	215
	0.023 µM	289	168
	0.07 µM	257	226

^1^ The higest concentration of inhibitor tested during efflux experiments was used to determine the percentage of intracellular l-lactic acid increase i.e., atorvastatin 300 µM, simvastatin acid 300 µM and loratadine 250 µM and effects of pretreatment on inhibition potential were compared (* *p* < 0.02).

## Supplementary Materials

The following are available online at www.mdpi.com/1999-4923/9/4/42/s1, Figure S1: Relative mRNA expression levels of drug-transporters in SkMC following pretreatment with statins at different concentrations (0.2 and 2 µM of atorvastatin, 0.2 and 2 µM of simvastatin acid, 0.2 and 2 µM of rosuvastatin). Gene expression levels were normalized using GAPDH as an housekeeping gene and vehicle-treated SkMC were used as reference. OATP1B1 was also investigated and no expression of OATP1B1 was detected in any SkMC samples, Table S1: Summary of HPLC analytical method conditions for the quantification of statins and loratadine (flow rate of 1.0 mL/min). Details pertaining to Western blotting method are also available.
