# Supplementary material for: Study of Statin- and Loratadine-Induced Muscle Pain Mechanisms Using Human Skeletal Muscle Cells
Source: Pharmaceutics. 2017 Oct 10;9(4):42. doi: 10.3390/pharmaceutics9040042 (PMC5750648; doi:10.3390/pharmaceutics9040042)
Supplement: Supplementary file 1 [file pharmaceutics-09-00042-s001.pdf]

Supplementary Materials: Study of Statin- and Loratadine-Induced Muscle Pain Mechanisms Using Human Skeletal Muscle Cells

Yat Hei Leung, Jacques Turgeon and Veronique Michaud

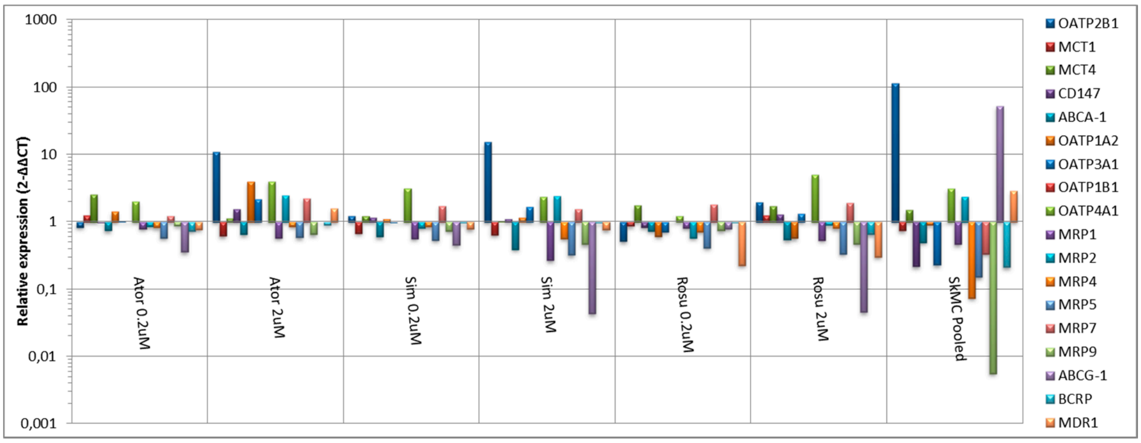

Figure S1. Relative mRNA expression levels of drug-transporters in SkMC following pretreatment with statins at different concentrations (0.2 and 2 µM of atorvastatin, 0.2 and 2 µM of simvastatin acid, 0.2 and 2 µM of rosuvastatin). Gene expression levels were normalized using GAPDH as an housekeeping gene and vehicle-treated SkMC were used as reference. OATP1B1 was also investigated and no expression of OATP1B1 was detected in any SkMC samples.

Table S1. Summary of HPLC analytical method conditions for the quantification of statins and loratadine (flow rate of 1.0 mL/min).

Compound	Proportion of Mobile Phase: 10 mM Ammonium Formate pH 3: Acetonitrile (v:v)	Internal Standard	Monitored UV Wavelength (nm)
Atorvastatin	53:47	Naproxen	250
Loratadine	60:40	Lovastatin	248
Rosuvastatin	53:47	Naproxen	243
Simvastatin hydroxyl acid	53:47	Loratadine	248
Simvastatin lactone	60:40	Loratadine	246

Western blot analysis

Total protein content was extracted from SkMC. Cells were lysed in a sample buffer containing 1% SDS/0.2N NaOH or commercially available cell lysis buffer (Cell Signaling Technology, Danvers, MA, USA). Protein concentration of the protein lysate was determined by Pierce BCA Protein Assay Kit (Thermo Fisher Scientific, Walthman, MA, USA) with bovine serum albumin as a standard, following the manufacturer's recommendations. For the Western blot analysis, samples were denatured at 100°C for 5 minutes in a loading buffer containing 50 mM Tris-HCL, 100 mM DTT, 2% SDS, 0.1% bromophenol blue, and 10% glycerol and separated in 5% stacking and 10% SDS polyacrylamide gels. Proteins were transferred by electrophoresis onto a pure nitrocellulose membrane (BioTrace, Onenhuga, Auckland, New Zealand). Membranes were blocked with TBS containing 0.05% Tween 20 (TBS/T) and 5% dry milk. Membranes were washed with TBS/T and incubated with primary antibody mouse anti-GAPDH (Sigma-Aldrich, St. Louis, MO, USA) (diluted 1:10000), mouse anti-MCT1 (diluted 1:500), or rabbit anti-MCT4 (Santa Cruz Biotechnology, Dallas, TX, USA) (diluted 1:500). Then, membranes were washed and incubated with secondary antibody

conjugated with horseradish peroxidase goat-anti mouse (diluted 1:5000) or goat-anti rabbit (Santa Cruz Biotechnology, Dallas, TX, USA) (diluted 1:5000). Bands were visualized on Hyblot CL autoradiography film (Denville Scientific, Holliston, MA, USA) with a standard enhanced chemiluminescence developing solution (GE Healthcare, Mississauga, ON, Canada).
